# Current Concepts in the Evaluation and Management of Type II Superior Labral Lesions of the Shoulder

**DOI:** 10.2174/1874325001812010331

**Published:** 2018-07-31

**Authors:** William A. Hester, Michael J. O’Brien, Wendell M.R. Heard, Felix H. Savoie

**Affiliations:** Tulane University School of Medicine, Department of Orthpaedic Surgery, New Orleans, LA 70112, USA

**Keywords:** Biceps tenodesis, Biceps tenotomy, SLAP lesion, SLAP repair, SLAP tear, Shoulder

## Abstract

**Background:**

Superior labrum tears extending from anterior to posterior (SLAP lesion) are a cause of significant shoulder pain and disability. Management for these lesions is not standardized. There are no clear guidelines for surgical versus non-surgical treatment, and if surgery is pursued there are controversies regarding SLAP repair versus biceps tenotomy/tenodesis.

**Objective:**

This paper aims to briefly review the anatomy, classification, mechanisms of injury, and diagnosis of SLAP lesions. Additionally, we will describe our treatment protocol for Type II SLAP lesions based on three groups of patients: throwing athletes, non-throwing athletes, and all other Type II SLAP lesions.

**Conclusion:**

The management of SLAP lesions can be divided into 4 broad categories: (1) nonoperative management that includes scapular exercise, restoration of balanced musculature, and that would be expected to provide symptom relief in 2/3 of all patients; (2) patients with a clear traumatic episode and symptoms of instability that should undergo SLAP repair without (age < 40) or with (age > 40) biceps tenotomy or tenodesis; (3) patients with etiology of overuse without instability symptoms should be managed by biceps tenotomy or tenodesis; and (4) throwing athletes that should be in their own category and preferentially managed with rigorous physical therapy centered on hip, core, and scapular exercise in addition to restoration of shoulder motion and rotator cuff balance. Peel-back SLAP repair, Posterior Inferior Glenohumeral Ligament (PIGHL) release, and treatment of the partial infraspinatus tear with debridement, PRP, or (rarely) repair should be reserved for those who fail this rehabilitation program.

## 1 INTRODUCTION

Tears of the superior labrum extending from anterior to posterior (SLAP) are a cause of significant shoulder pain and disability. Initially described by Andrews *et al.* in 1985 [1] and later classified by Snyder *et al.* in 1990 [2], the treatment of SLAP lesions continues to be a topic of controversy. This paper will give a brief background of SLAP lesions and define the authors’ treatment algorithm for Type II SLAP lesions.

## 2 ANATOMY OF THE LABRUM

The labrum increases the surface area of glenoid articulation with the humeral head, helping to maintain glenohumeral stability. The labrum serves as the attachment site of the long head of the biceps tendon, and the superior, middle, and inferior glenohumeral ligaments are continuous with the labrum.

The superior and inferior portions of the glenoid labrum differ. Grossly, the superior portion of the glenoid labrum is similar in appearance to the meniscus of a knee in having a triangular cross-sectional shape [3, 4]. The inferior portion has been described as having a more rounded appearance at the free edge along the glenoid face [3]. Histologically, some studies have shown that the labrum is a fibrocartilaginous structure [5] while others have shown it to be fibrous tissue with a portion of fibrocartilage [3, 4].

Vascularity of the labrum arises from a network of vessels originating from the suprascapular artery, the circumflex scapular branch of the subscapular artery, and the posterior circumflex humeral artery. The vascular penetration of the labrum is more predominant at the peripheral attachment to the joint capsule [3], with less vasculature present in the central zone [5]. It has been shown that this vascular network diminishes greatly in the first year of life and continues to diminish throughout the remainder of life [5]. Furthermore, no vessels are known to arise from the glenoid to supply blood to the labrum [3]. Cooper *et al.* [3] also showed that the anterosuperior portion of the labrum is less vascular than the rest of the labrum.

## 3 CLASSIFICATION OF SLAP LESIONS

Snyder *et al.* [2] first described the classification of SLAP lesions as Types I-IV. Type I lesions represent a degenerative mechanism characterized by both fraying of the superior glenoid labrum with the peripheral labrum and the biceps anchor still firmly attached to the glenoid. Type II lesions also show fraying, but the labrum and biceps anchor are stripped from the superior glenoid. Type III consists of a bucket-handle lesion of the superior labrum. Type IV is also a bucket-handle lesion, but there is an extension into the biceps tendon. SLAP lesions are further characterized into 10 subtypes based on associated instability and posterior extension. These are not used in the literature as often as the Snyder classification [6, 7].

## 4 MECHANISM OF INJURY

**Fig. (1):**
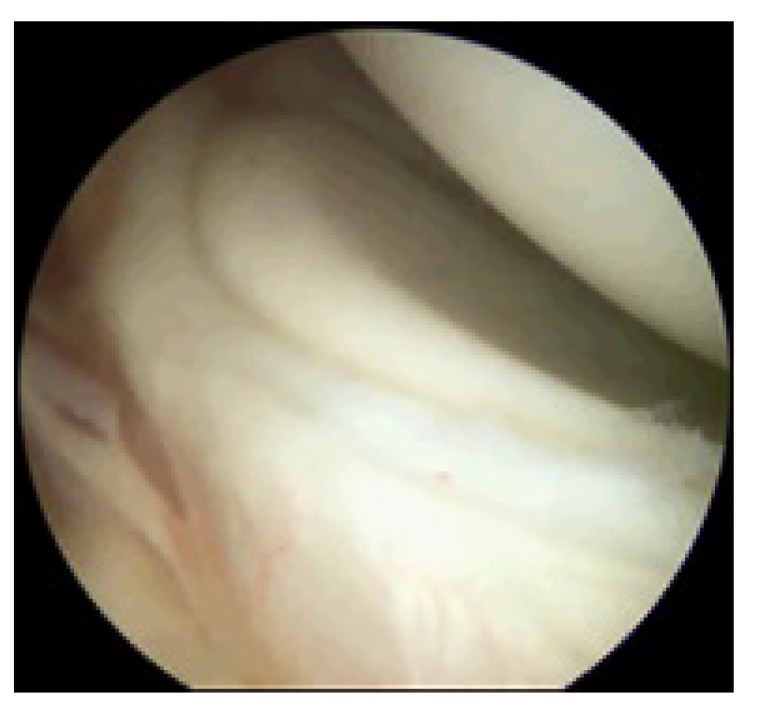
Right shoulder viewed from the posterior portal in the lateral decubitus position showing a peel-back lesion.

A number of mechanisms have been theorized to cause SLAP lesions. Bey *et al.* [8] created Type II SLAP lesions by causing a biceps tendon traction injury from inferior subluxation. Clavert *et al.* [9] used a cadaver model to suggest a shearing mechanism from a fall onto an outstretched hand. A number of studies have shown that repetitive overhead throwing is associated with the development of SLAP lesions [10-14]. Burkhart and Morgan [14] proposed the peel-back mechanism in baseball players, in which abduction and external rotation cause a rotational force at the biceps tendon; this creates a torsional force that is transmitted through the tendon, peeling the posterior labrum from the glenoid (Fig. **1**).

## 5 DIAGNOSIS

Clinical diagnosis of SLAP lesions can be difficult. Patients may complain of anterior shoulder pain, clicking, or a sense of instability. There are multiple physical examination maneuvers described in the literature: active compression test [15], Speed test [16], anterior slide test [17], crank test [18], and the Yergason test [19]. Hegedus *et al.* [20] and Parentis *et al.* [21] showed no single test had sufficient sensitivity and specificity for the consistent diagnosis of SLAP lesions. The highest sensitivity (67%) was seen with the active compression test, but specificity was only 37% [15]. The highest specificity was seen with the Yergason test (95%), which showed a sensitivity of 12% [20]. It should be mentioned that, in the authors’ opinion, the sensitivity and specificity were based on MRI analysis at a time when MRI and MRA routinely “overcalled” SLAP lesions. Thus, the exams may well have been much more sensitive and specific if only truly unstable SLAP lesions were included.

## 6 AUTHORS’ SPECIFIC EXAM TECHNIQUE

We prefer to use the SLAP test [22] to check for anterior superior shifting, and the Modified DLS test [23] to detect a labral click to determine if the SLAP lesion is unstable. The presence of posterior pain (due to internal impingement of the infraspinatus on the rim of the glenoid) on either of these examination techniques is a specific indication for labral repair, regardless of how one treats the biceps. The Whipple test with and without manual scapular retraction can provide information on the patient's ability to compensate for the injury. Directional load and shift testing, a much more subtle examination test, will often reproduce the symptoms. It is the authors experience that truly unstable SLAP lesions will always have a positive examination finding.

## 7 DIAGNOSTIC IMAGING

MRI-arthrography has been reported to have a sensitivity as high as 96% and specificity as high as 85% [24-26]. However, these imaging studies are uniformly poor in differentiating normal age-related deterioration from truly unstable labral lesions, and call into question any MRI findings taken in isolation when determining surgical indications. The extreme anatomic variability of the superior labrum and the inability to determine whether it is truly symptomatic means that each report must be correlated with the patient’s history and physical examination to be helpful. Even diagnostic arthroscopy gives mixed results in terms of inter-observer and intra-observer reliability [27-29].

## 8 INITIAL MANAGEMENT: REHABILITATION

Fedoriw *et al.* [30] have shown that about two-thirds of SLAP patients respond to rehabilitation focused on postural correction and balancing exercises in professional baseball players. In our clinic, the initial management of all SLAP injuries involves three basic principles:

1. Decrease inflammation *via* cryotherapy, medications, and/or injections. 2. Postural correction *via* scapular retraction exercises, posture bracing and taping, and biofeedback exercises. 3. Balanced rotator cuff rehabilitation and proprioceptive neuromuscular rehabilitation exercises to return to function, while always monitoring the scapular position.

In the throwing athlete, hip range of motion, abductor strength, and core exercises are emphasized and corrected as well as the three principles above.

## 9 SURGICAL TREATMENT

The discussion on surgical treatment will focus on Type II SLAP lesions. Treatment of SLAP lesions remains controversial. The patient’s age, activity level, occupation, expectations, and workers’ compensation status are all factors in the decision-making process. The preoperative history and examination is a critical part of the decision-making process. The management of the throwing athlete also represents a unique category, and the examination, non-operative, and operative treatment of these athletes should be considered separately. Findings consistent with biceps involvement (a positive Speed and Yergason test, tenderness to palpation over the bicipital groove or subpectoral area, and pain radiating into the biceps muscle belly) may indicate that the patient may also require a biceps tenodesis or tenotomy instead of, or in conjunction with, the SLAP repair.

## 10 GROUP 1 (NON-THROWING ATHLETES)

**Fig. (6):**
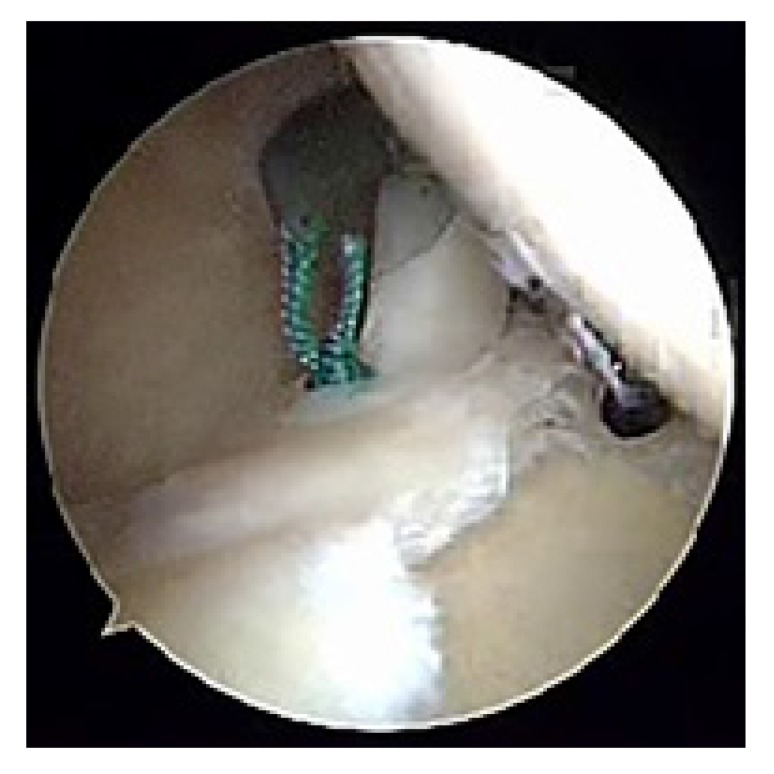
Right shoulder (lateral decubitus position) as viewed from the posterior portal with SLAP repair completed.

In this group of patients, the history and examination are critical. The history usually includes a significant and easily recalled trauma. The patient will also complain of shifting, clicking, or popping in the shoulder with certain movements. A critical component is the presence of posterior shoulder pain, usually indicating humeral head mal-tracking, which allows internal impingement of the infraspinatus on the posterior superior glenoid. Similarly, the presence of a positive SLAP test and Whipple test indicate anterosuperior instability. In these patients Fig. (**2**), the labrum should be repaired. In patients with biceps pain when palpating deep to the pectoralis major tendon, we add in a biceps tenodesis or tenotomy but always repair the labrum with a posterior superior and anterior superior anchor. The posterior anchor is placed *via* the port of Wilmington and the sutures passed in standard techniques at 10 and 11 o’clock, as well as 11 and 12 o’clock Fig. (**3**). We have found it much easier to pass the 12 o’clock suture by using a spinal needle *via* the Neviaser portal, shuttling it with a Polydioxanone Suture (PDS) (Ethicon, a division of Johnson and Johnson, New Jersey) Fig. (**4**). We always use mattress sutures for posterior SLAP repairs to avoid knot impingement. The other option is knotless anchors. In my revision cases where the first surgeon used simple sutures with knots they tend to impinge on the undersurface of the infraspinatus in the abducted, externally rotated position. The anterior superior anchor is placed just anterior to the biceps, the superior glenohumeral ligament is repaired with a mattress suture, and a biceps tenodesis is performed if the tendon appears abnormal Fig. (**5**). If the biceps tendon is normal on exam, imaging, and arthroscopic inspection both inside the joint and in the sub-deltoid space, we usually leave it alone (Fig. **6**).

## 11 GROUP II (OVERUSE INJURIES)

**Fig. (7):**
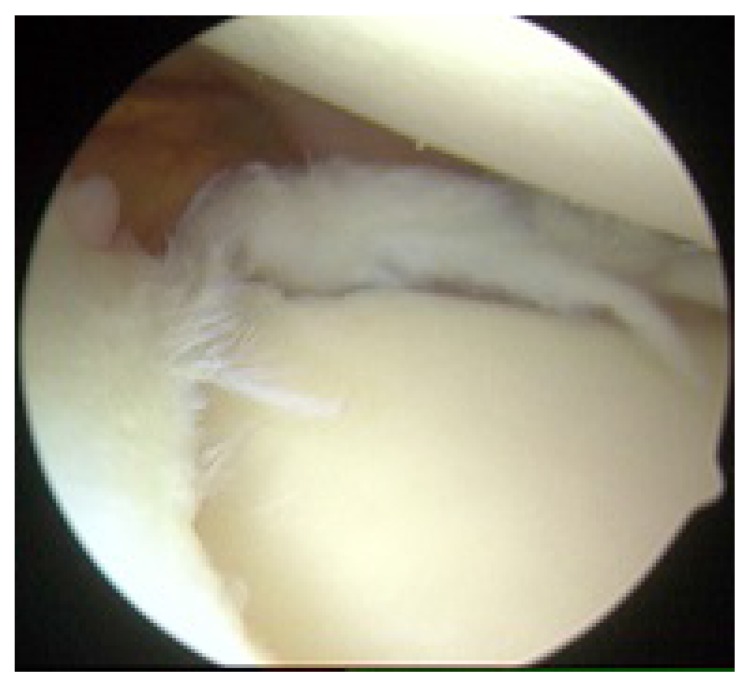
Right shoulder with patient in the lateral decubitus position as viewed from the posterior portal. The labrum and biceps anchor have been stripped from the superior glenoid consistent with a Type II SLAP lesion.

This group is far more common, and it is also the group that usually responds to non-operative management. In this group, there is usually a history of some type of injury, but the history may be inconsistent with a history of subluxation event. The physical examination will often be generally painful, but the SLAP test, modified DLS test, and posterior impingement tests will all be negative for clicking or shifting. Additionally, the Whipple test will become negative with scapular retraction. In these patients, the diagnosis of SLAP lesion will have often been made *via* MRI. If the patient goes to surgery, we would usually perform a biceps tenodesis along with debridement of the superior labrum, but no SLAP repair would be performed. There are multiple effective tenodesis techniques, and we have not found a significant difference between any of them (Fig. **7**).

## 12 GROUP III (THROWING ATHLETES)

**Fig. (8):**
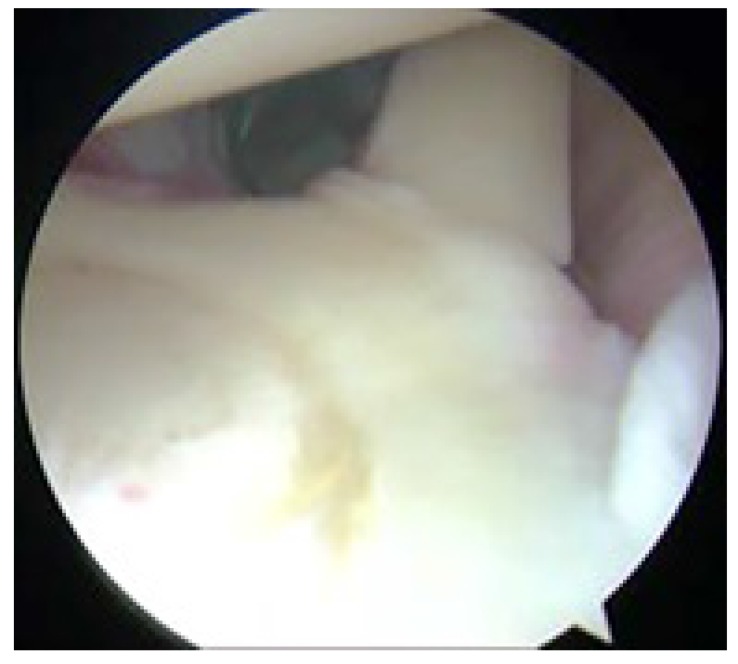
Left shoulder with the patient in the lateral decubitus position, view from the posterior portal. The arm is abducted and externally rotated, showing the posterior superior labrum separating from the superior glenoid as seen with a peel-back lesion.

In this group, it is vital for the patient to have completed an effective non-operative treatment program that includes hip stretching and strengthening, core exercises, scapular rehabilitation, positional correction, as well as shoulder and elbow exercises. In those throwers in whom this extensive rehabilitation program fails surgery may be contemplated. In the throwing athlete, the initial step is a comparative exam under anesthesia for motion of the shoulder. The arc of combined ER to IR should be at least equal side-to-side (total arc of motion). One would expect the throwing shoulder to have increased external and decreased internal rotation. Differences in subluxation should also be evaluated on the side to side examination. Arthroscopic repair in these patients should be meticulous and as minimally disruptive as possible. Diagnostic arthroscopy will usually show a small area of scarring in the posterior inferior glenohumeral ligament (PIGHL), a posterior superior labrum that is irregular and is hypermobile on arthroscopic DLS testing, and may show mild anterior subluxation. The infraspinatus will likely show fraying and partial tearing. The initial part of the surgery is to palpate and (rarely) release the PIGHL contracture. This release, if performed, is limited to about 1 cm in length. The next step is to recheck the posterior superior labrum mobility. If internal impingement still occurs, then a posterior superior labrum repair is performed as previously described. In the throwing athlete we keep the suture and anchor posterior, away from the biceps. When the arthroscopic DLS test is repeated we expect to find that the internal impingement no longer occurs. If it still impinges, an absorbable suture is carefully placed in the anterior capsule at the 3 o’clock position to add to the stability of the shoulder, after which we gently debride the infraspinatus tear. We usually also inject this area with leukocyte-rich, platelet-rich plasma under direct visualization (Fig. **8**).

## 13 POST-OPERATIVE REHABILITATION

The patients are all placed in an abduction sling for 4-6 weeks. Scapular retraction exercises begin immediately. Active motion is initiated within a pain-free range at week two and progresses very carefully, as tolerated, while keeping the scapula retracted at all times. At 4-6 weeks post-op, more aggressive rehabilitation is initiated and progressed as tolerated.

## 14 RESULTS

Outcomes of Type II SLAP repairs have been reported as good to excellent in 65-97% of patients [31-34]. It is important to note that the outcome of a SLAP repair is highly dependent on the pre-injury activity level of the patient and that differences have been noted between overhead athletes and non-athletes. Additionally, when concurrent intra-articular pathology is treated surgically with a SLAP lesion, the outcomes are not as good. Friel *et al.* [31] evaluated 46 patients who underwent arthroscopic SLAP repair. Several patients had concurrent Bankart repair, subacromial decompression, distal clavicle excision, and intra-articular debridement. Overall, 79% of patients reported excellent results subjectively. Of 13 overhead college athletes, 7 (54%) returned to their pre-operative level of play, including only 1 of 4 collegiate tennis players. Low rates of return to sport after SLAP repair have been seen in multiple studies [32, 33]. Neri *et al.* showed a 57% return to high performance overhead competition, which was consistent with previous studies [35, 36]. Concomitant partial articular-sided tendon avulsion lesions that underwent debridement were identified as an independent risk factor for the inability to return to sport [35]. A retrospective study by Van Kleunen *et al.* [37] looked at 17 overhead throwing athletes undergoing repair of both a Type II SLAP lesion and infraspinatus tear. Their study demonstrated a 35% rate of return to a preinjury sporting level in this group of athletes. Brockmeier *et al.* [38] evaluated 34 athletes (28 overhead athletes) who had a labral repair and reported a 74% rate of return to pre-injury level of participation. It is the authors’ opinion that the vast majority of SLAP lesions do not require surgery. Proper non-operative management should be effective at least 70% of the time. Thus the indications for SLAP repair are symptomatic instability with a positive examination that has failed to respond to conservative therapy. This usually represents less than 5% of the authors’ surgical practice.

## 15 COMPLICATIONS

Complications after SLAP repair include stiffness, pain at rest, painful range of motion, nerve injury, and mechanical symptoms. Postoperative stiffness has been reported as the most common cause of SLAP repair failures [39, 40]. Revision surgery may include biceps tenodesis, loose body removal, removal of suture, manipulation, capsular release, and revision of the SLAP repair, the last of which is rarely indicated. Arthroscopy for revision surgery will often show partial tears of the long head of the biceps tendon with rotator interval inflammation, suggesting that the original SLAP repair was not necessary [41, 42]. McCormick *et al.* [41] showed significant improvement in American Shoulder and Elbow Surgeons (ASES), Single Assessment Numeric Evaluation (SANE), and Western Ontario Shoulder Instability Index (WOSI) scores with biceps tenodesis after reported poor outcomes of Type II SLAP lesions treated with repair.

## 16 DISCUSSION

Indications for SLAP repair are not well supported in the literature at this time. In a systematic review of SLAP repairs by Kibler and Sciascia [43], 26 manuscripts were reviewed, and 54% of these did not report an indication for repair. The remaining papers reported “anatomic alterations such as labral/biceps tear or separation or excessive labral mobility” as indications for SLAP repair. However, some authors have described their indications for surgical intervention for Type II SLAP lesions. Some authors recommend repair for patients younger than 35-40 years old and biceps tenotomy or tenodesis for patients older than 35-40 years old [44, 45]. Others recommend repair with “biceps stabilization” in Type II SLAP lesions [46, 47]. Wilk *et al.* [46] also recommend concurrent treatment of associated rotator cuff lesions or glenohumeral instability.

Provencher *et al.* [48] prospectively studied repair of isolated Type II SLAP lesions in 179 active duty soldiers who are required to do heavy overhead work as part of their daily life. Surgical indications were described in their study as clinical exam and magnetic resonance arthrogram with evidence of Type II SLAP lesion. Approximately half of the patients included in this study had traction injuries due to heavy lifting as an inciting event. Surgery provided a statistically significant improvement in range of motion as well as ASES, SANE, and WOSI post-operative scores at approximately 2.5 year follow-up. They recognized a failure rate of 36.8% (66 patients) with the only preoperative risk factor identified as a patient age of greater than 36 years. The revision was performed in 50 of these patients and included biceps tenodesis (42 patients), biceps tenotomy (4 patients), or debridement (4 patients). None of their patients required revision SLAP repair.

Boileau *et al.* [49] compared biceps tenodesis and SLAP in 25 patients and found that 40% of the patients in the repair group were satisfied, with only 20% being able to return to their previous level of activity. In the tenodesis group, 93% were satisfied and 87% returned to their previous sporting level. Of note is the mean age in the SLAP repair group at 37 years old and at 52 years old in the biceps tenodesis group. This seems to support biceps tenodesis in an older population with Type II SLAP lesions. Ek *et al.* [45] retrospectively compared biceps tenodesis and SLAP repair in 35 patients and found that 76% of biceps tenodesis and 60% of SLAP repair patients returned to the previous level of sporting activity. Their indications for repair were age less than 35 years *and* a healthy appearing labrum at the time of arthroscopy.

Throwing athletes, especially baseball players, with a Type II SLAP lesion are considered to be in their own category. Studies have shown that repair of SLAP lesions in this group does not have the same good to excellent results as the rest of the population with this tear [35-38]. In this population, glenohumeral internal rotation deficit (GIRD) caused by contracture of the PIGHL leads to SLAP lesions and rotator cuff pathology, and was noted with the first descriptions of SLAP lesions [1, 2]. Van Kleunen *et al.* [37] showed that overhead throwing athletes undergoing repair of a Type II SLAP lesion and infraspinatus tear had a 35% rate of return to a preinjury sporting level. They found that repair of an infraspinatus tendon tear with a suture anchor was more likely to cause the athlete to be unable to return to sport than repair with PDS suture. They also found the release of the PIGHL trended toward higher Kerlan-Jobe Orthopaedic Clinic Overhead Athlete Shoulder and Elbow scores (a validated scoring system for functional status of overhead throwing athletes) [36]. As a result of this, our preferred method is to minimally release the PIGHL, perform a SLAP repair, and use either a single PRP injection or a PDS suture for repair of the infraspinatus. We believe this minimizes the amount of iatrogenic trauma to the shoulder at the time of surgery.

## CONCLUSION

In our opinion, repair of the superior labrum requires three findings: (1) a history of a significant subluxation event; (2) symptoms of instability consistent with the inciting event; and (3) a physical examination test that confirms the instability pattern and reproduces the symptoms, ie the DLS or slap test, inferior subluxation or positive load and shift testing that reproduces the symptoms. In patients who fit these three criteria, repair can be extremely effective with or without biceps tenodesis.

The diagnosis and management of SLAP lesions remain controversial. A detailed history and physical examination are more valuable than imaging. Non-operative management focused on scapular rebalancing is often effective. Surgery should include repair of the SLAP lesion if the history and physical examination are consistent with instability. We prefer a tenotomy or tenodesis if there is no concern for instability, and will do both procedures if indicated by history, symptoms, and examination. Treatment of overhead throwing athletes, such as baseball players, with peel-back SLAP lesions should be focused on aggressive rehabilitation. Surgery, if performed, should be as minimal as possible to improve their chances of both return to sport and preinjury activity level.

## Figures and Tables

**Fig. (2):**
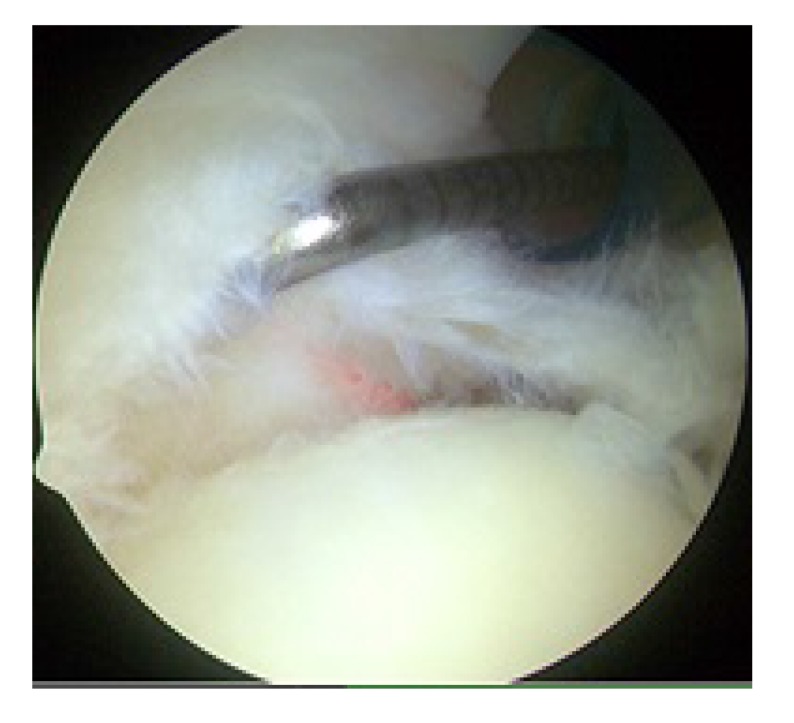
Right shoulder with the patient in the lateral decubitus position showing a Type II SLAP lesion as viewed from the posterior portal with probe coming in from the anterior portal. The biceps anchor attachment has been disrupted.

**Fig. (3):**
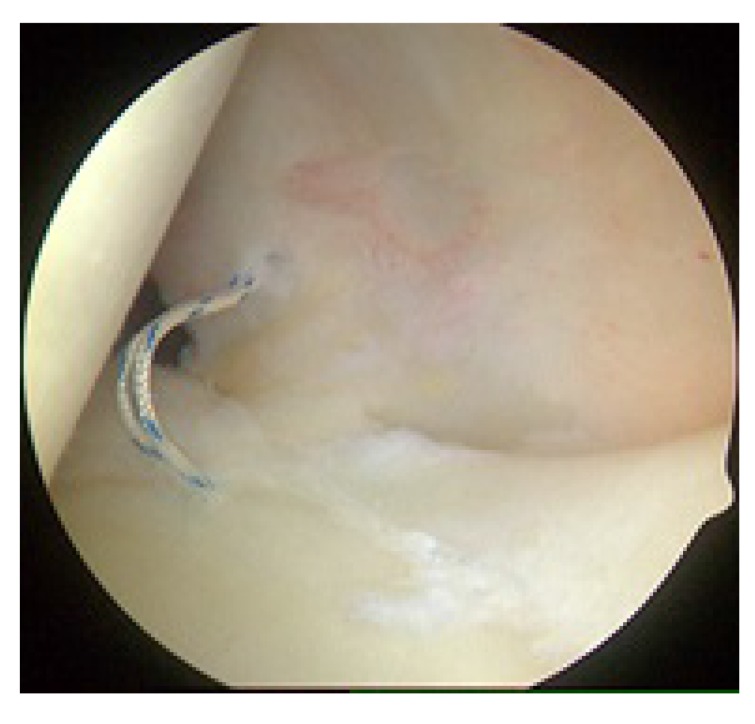
Right shoulder with the patient in the lateral decubitus position, view from the anterior portal. The initial suture anchor for SLAP repair has been placed between the 10 o’clock and 11 o’clock position on the glenoid through the port of Wilmington.

**Fig. (4):**
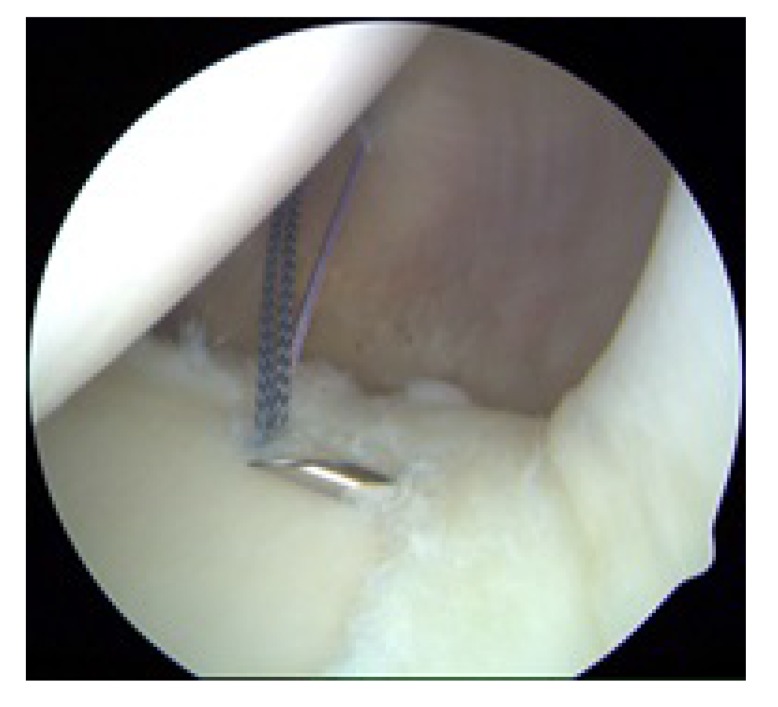
Right shoulder with the patient in the lateral decubitus position as viewed from an anterior portal. A 12 o’clock suture will be passed by shuttling a PDS suture using a spinal needle *via* the Neviaser portal.

**Fig. (5):**
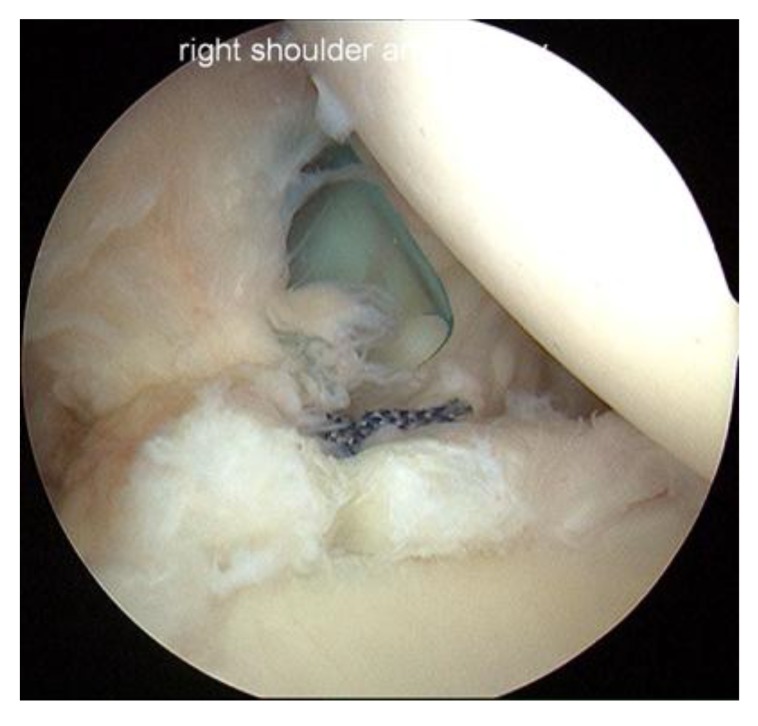
Right shoulder (lateral decubitus position) as viewed from the posterior portal with SLAP repair and biceps tenotomy completed. The biceps can be left in the tenotomized state, or a tenodesis may be performed with the surgeon’s preferred technique.
